# Synthesis of a single-crystal Fe_2_O_3_ nanowire array based on stress-induced atomic diffusion used for solar water splitting

**DOI:** 10.1098/rsos.172126

**Published:** 2018-03-14

**Authors:** Yiyuan Xie, Yang Ju, Yuhki Toku, Yasuyuki Morita

**Affiliations:** Department of Mechanical Science and Engineering, Graduate School of Engineering, Nagoya University, Nagoya 464-8603, Japan

**Keywords:** Fe_2_O_3_ nanowire, solar water splitting

## Abstract

In this study, we successfully fabricated a single-crystal Fe_2_O_3_ nanowire array based on stress-induced atomic diffusion and used this array as the photoelectrode for solar water splitting. With the surface polishing treatment on the sample surface, the density of the Fe_2_O_3_ nanowire array reached up to 28.75 wire µm^−2^ when heated for 90 min at 600°C. The photocurrent density of the optimized sample was 0.9 mA cm^−2^ at 1.23 V versus a reversible hydrogen electrode in a three-electrode system under AM 1.5 G illumination. The incident photon-to-electron conversion efficiency was 6.8% at 400 nm.

## 1. Introduction

A clean and sustainable energy resource is indispensable for human beings because of the massive fossil fuel consumption that comes along with industrial development. Hydrogen is a suitable candidate. However, traditional production methods use fossil fuels to obtain it, which would not eventually solve the energy crisis. Solar water splitting is a clean and sustainable production method to produce hydrogen, because it uses only sunlight and water, which are the two most abundant natural resources. The anode in a solar water splitting system can be a semiconductor material with a suitable bandgap, while the cathode can be a metallic material. Both the anode and cathode are placed in water or an electrolyte solution. When the photoanode is illuminated by sunlight, the photons with an energy larger than the bandgap of the semiconductor will excite the electrons from the valence band to the conduction band. The excited electrons will then move to the cathode and react with the electrolyte solution to produce hydrogen [1].

Several promising materials, including WO_3_ [2], BiVO_4_ [3], TiO_2_ [4] and Fe_2_O_3_ [5], have been studied as the electrode materials for solar water splitting. These candidates must satisfy some requirements, such as a small semiconductor bandgap, theoretical maximum solar-to-hydrogen conversion efficiency, durability in aqueous environments and low cost. Among these materials, Fe_2_O_3_ seems to satisfy all the requirements of the photoanode: a small bandgap of 2.1 eV, a high theoretical solar-to-hydrogen conversion efficiency of 15.3% [6], and excellent stability under alkaline conditions. Iron is also the fourth most common element on the Earth (6.3% by weight) [7]. However, some disadvantages limit the solar-to-hydrogen efficiency of Fe_2_O_3_. These disadvantages include a very short excited-state lifetime [8], a short hole diffusion length of 2–4 nm [9], and poor electrical conductivity. Accordingly, several strategies were studied to overcome these disadvantages. For example, doping another material on the Fe_2_O_3_ is a popular method of improving the photocatalytic performance of the Fe_2_O_3_ photoanode because it could improve the hole transport [10,11] and alter the bandgap [12]. A wide range of dopants, such as Pt [13,14], Cu [15], Si [16] and Sn [17], have also recently been used.

Meanwhile, the fabrication of a nanostructured Fe_2_O_3_ photoanode, such as nanorod, nanoparticle and nanotube, is another viable strategy to improve the photoelectrochemical performance. Compared with a bulk material, a nanostructured material could provide a significant enlargement of the material surface area, which could increase the absorption of sunlight and the contact surface between the photoanode and the electrolyte [18]. A nanostructure is also helpful in overcoming the short-hole diffusion length of Fe_2_O_3_, because it could reduce the necessary path length of the hole transport [19]. Similar studies on the Fe_2_O_3_ nanostructures were reported. For example, Gurudayal *et al*. reported a haematite nanorod photoanode with a photocurrent of 0.45 mA cm^−2^ at 1.23 V versus a reversible hydrogen electrode (RHE) [20]; Sivula *et al*. reported an Fe_2_O_3_ nanoparticle photoanode with a photocurrent of 0.56 mA cm^−2^ at 1.23 V versus RHE [21]; and Momeni *et al*. fabricated an Fe_2_O_3_ nanotube photoanode with a photocurrent of 0.35 mA cm^−2^ at 1.23 V versus RHE [22].

Although the photoelectrochemical performance could be improved using the doping method, increasing the efficiency of a pure Fe_2_O_3_ nanostructure remains a challenge. Compared to other nanostructures, a nanowire array could provide a larger surface area because of the high aspect ratio, which could not only absorb more light than other nanostructures (e.g. nanorods, nanoparticles and nanotubes), but also increase the photoelectrode–electrolyte interface area, thereby enhancing the chemical reaction of water splitting. Meanwhile, the nanowire structure could reduce the diffusion distance of photogenerated minority carriers from the centre to the nanowire surface because of the small diameter. Therefore, photogenerated electron–hole pairs could efficiently separate before recombination in the nanowire structure, which could eventually increase the solar water splitting efficiency. We recently proposed a method to fabricate a high-density polycrystalline Fe_2_O_3_ nanowire array based on oxidation-assisted stress-induced atomic diffusion by introducing a water vapour environment [23]. The present study demonstrates a new method of fabricating an extremely high-density single-crystal Fe_2_O_3_ nanowire array based on stress-induced atomic diffusion with surface polishing treatment. A single-crystal nanostructure is believed to reduce the charge carrier loss because of the electron–hole recombination [24]. The surface polishing treatment could enhance the surface oxidation process, thereby increasing the driving force of atomic diffusion.

## 2. Experimental approach

### 2.1. Nanowire fabrication

A commercial iron plate with 99.95% purity was used as the substrate. The iron plate was 0.1 mm thick and measured 10 × 10 mm^2^. Two types of samples were prepared to investigate the effect of surface roughness on the nanowire growth. One was polished by a rasp, while the others were unpolished. The two types of samples were then put in alcohol and cleaned by an ultrasonic cleaner (AS ONE, ASU-2D). Subsequently, all samples were heated by a ceramic heater (SAKAGUCHI, SCR-SHQ-A) in air. The heating temperatures were changed to determine the optimized condition for the nanowire growth (table 1).

**Table 1.** Experimental conditions: different heating temperatures.

no.	heating time (min)	temperature (°c)	surface condition
1	90	500	unpolished
2		600	unpolished
3		700	unpolished
4		500	polished
5		600	polished
6		700	polished

### 2.2. Characterization

After the Fe_2_O_3_ nanowire array fabrication, scanning electron microscopy (SEM, JSM-7000FK) images were collected for the Fe_2_O_3_ nanowire arrays to study the morphology. A transmission electron microscope (TEM) observation was performed to obtain a high-resolution picture of the single nanowire and identify the crystalline structure of the Fe_2_O_3_ nanowires. An X-ray diffraction (XRD) analysis was also performed to study the structure and phase of the nanowire arrays.

### 2.3. Photocurrent measurements

The photocurrent density, incident photon-to-current conversion efficiency (IPCE), and stability measurements were conducted using a three-electrode system. A platinum wire was used as the counter electrode in a 1 mol l^−1^ NaOH solution electrolyte. A scanning potentiostat (ALS DY2325) was used to measure the photocurrent density. Light was provided by a solar simulator (SERIC XC-100EFSS) with the same spectrum as sunlight. The optical power density was 100 mW cm^−2^ at the test position equivalent to AM 1.5 G illumination. The IPCE measurements were performed using the same solar simulator with a monochromatic filter from 400 nm to 650 nm.

## 3. Results and discussion

### 3.1. Experimental results

**Figure 1.**
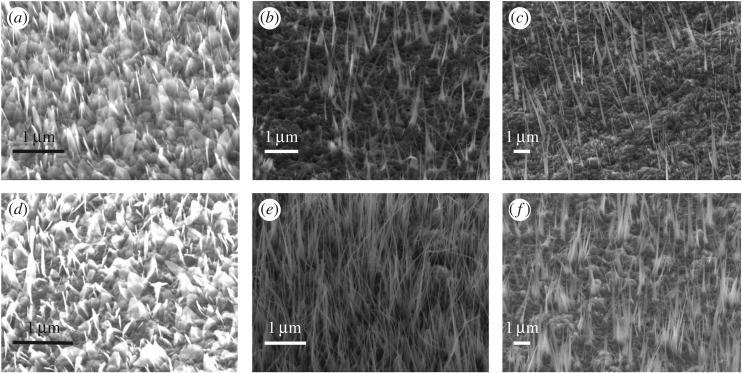
SEM micrographs of the Fe_2_O_3_ nanowire arrays obtained at different experimental conditions: (*a*) unpolished sample, 500°C; (*b*) unpolished sample, 600°C; (*c*) unpolished sample, 700°C; (*d*) polished sample, 500°C; (*e*) polished sample, 600°C; and (*f*) polished sample, 700°C.

**Figure 2.**
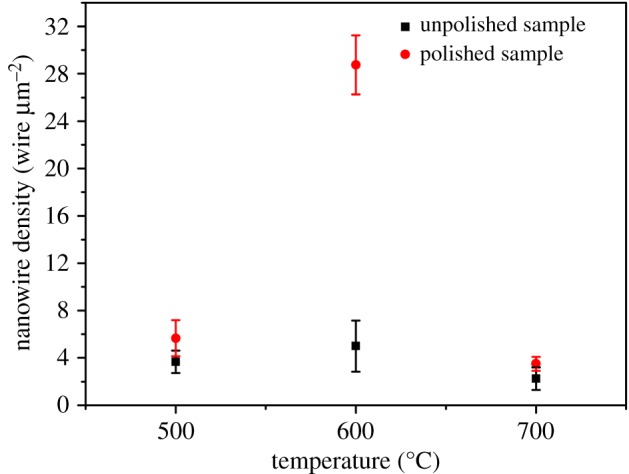
Statistical variation of density of the Fe_2_O_3_ nanowires obtained at different experimental conditions.

**Figure 3.**
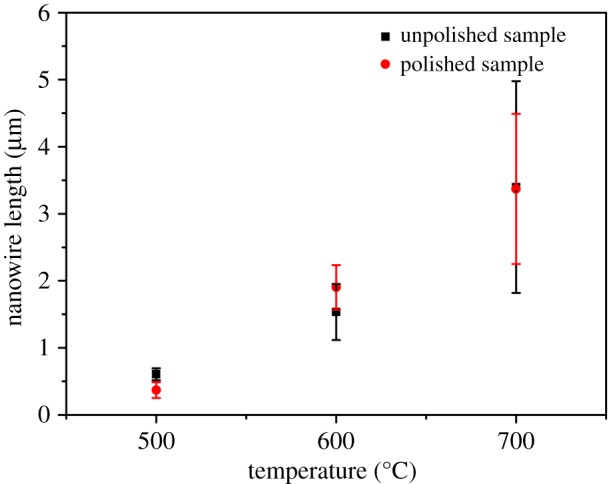
Statistical variation of length of the Fe_2_O_3_ nanowires obtained at different experimental conditions.

**Figure 4.**
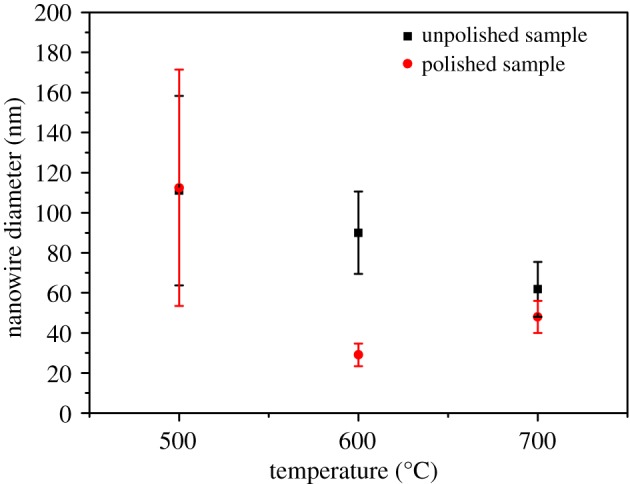
Statistical variation of diameter of the nanowires obtained at different experimental conditions.

Figure 1 shows the SEM image of the Fe_2_O_3_ nanowire arrays fabricated at 500°C, 600°C and 700°C with and without the surface polishing treatment. The SEM images showed that the sizes of the nanowire arrays fabricated under different temperatures were very different. Two types of nanowire shapes (i.e. wire- and leaf-shaped nanowires) were found in the sample heated at 500°C (figure 1*a,d*, respectively). The nanowire length for samples heated at 600°C was much longer than that heated at 500°C. Moreover, the diameter became smaller. The nanowires heated at 600°C on the unpolished sample had a long leaf shape, whereas those on the polished sample had a wire shape with an average diameter of 29 nm (figure 1*b,e*, respectively). Figure 1*c,f* shows the nanowires heated at 700°C on the unpolished and polished samples, respectively. The nanowires obtained from both the polished and unpolished samples had a long triangular shape. The average density, length and diameter of the nanowire arrays herein were evaluated based on the SEM observation results (figures 2–4, respectively).

Figure 2 depicts that the largest density of 28.75 wire µm^−2^ was found in the polished sample heated at 600°C for 90 min. This value was five times higher than the density of the unpolished sample (i.e. 5 wire µm^−2^) heated at the same condition. In the sample heated at 500°C, the density of the polished samples was higher than that of the unpolished samples. The nanowire arrays fabricated under all the experimental conditions were uniform, except for the unpolished sample heated at 700°C.

Meanwhile, one-dimensional nanostructures were reported to reduce the path length of the hole transport to improve the charge carrier recombination through a high aspect ratio and large surface area [25]. We compared the length and the diameter of the nanowires fabricated under different conditions (figures 3 and 4, respectively) to find the highest aspect ratio of the nanowire arrays. Consequently, the average length of the nanowires on both the polished and unpolished samples became longer with the increase in the heating temperature. The longest nanowires were obtained at 700°C from the unpolished sample of 3.37 µm. Furthermore, the nanowire length for the polished and unpolished samples was almost the same under the same temperature, indicating that the heating temperature was the key factor of the nanowire length. As for the diameter of the nanowire used for the photoanode in water splitting, a small diameter could reduce the diffusion distance of the photogenerated minority carriers from the centre to the semiconductor–electrolyte interface [26]. The smallest average diameter of 29 nm was obtained for the nanowires fabricated at 600°C for 90 min from the polished sample. The nanowire arrays fabricated at 500°C showed a wide range of diameters from 26 to 180 nm because the diameter of the wire-shaped nanowire was much smaller than that of the leaf-shaped nanowire.

### 3.2. Transmission electron microscope and X-ray diffraction observations

**Figure 5.**
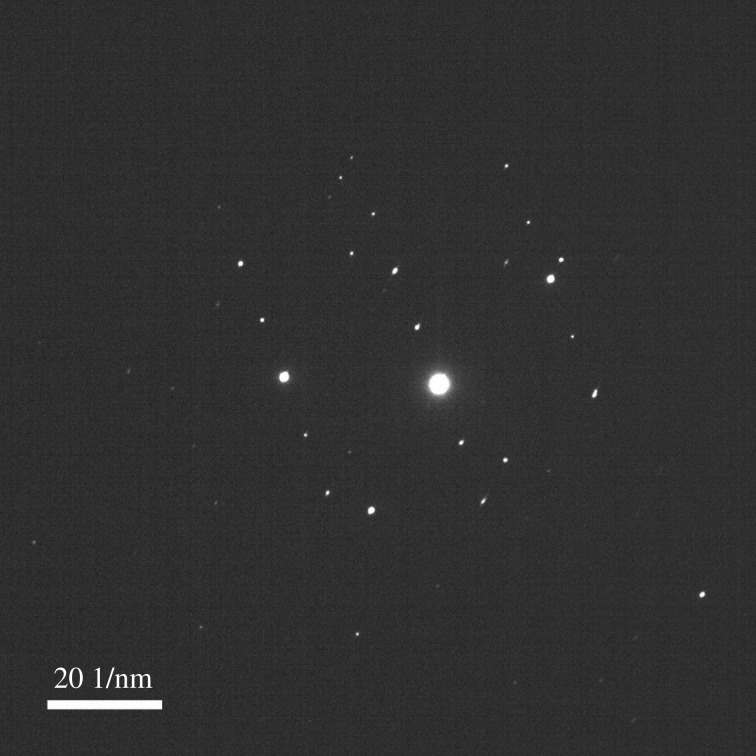
TEM diffraction pattern of the Fe_2_O_3_ nanowire arrays heated at 600°C for 90 min from the polished sample.

Figure 5 illustrates the TEM diffraction pattern of the single nanowire obtained from the polished sample heated at 600°C. The diffraction pattern image of the Fe_2_O_3_ nanowire confirmed that the nanowire fabricated under this condition was a single-crystal nanowire.

**Figure 6.**
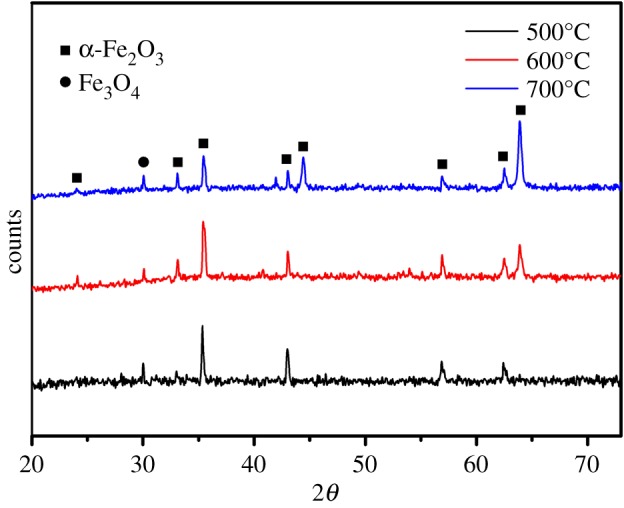
XRD patterns of the Fe_2_O_3_ nanowire arrays at different temperatures from the polished samples.

Representative XRD patterns of the nanowire arrays were obtained from the samples with the surface polishing treatment after heating at different temperatures of 500°C, 600°C and 700°C (figure 6). Most of the observed peaks can be indexed to the pure alpha-phase haematite, except for the peak at 30.18°, which was considered as that of Fe_3_O_4_. The iron plate surface is oxidized to the Fe_2_O_3_ layer when the heating temperature is over 500°C. However, information on the Fe_3_O_4_ layer under the Fe_2_O_3_ layer was collected from the result because the thickness of the formed Fe_2_O_3_ layer was smaller than the penetration depth in the XRD observation.

### 3.3. Photocurrent measurements

**Figure 7.**
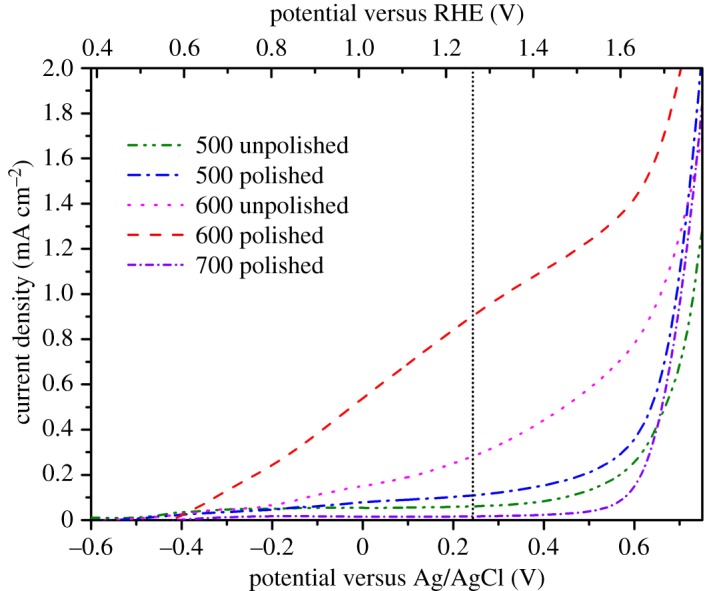
Photocurrents from the nanowire array anodes obtained at different experimental conditions.

Figure 7 shows the investigated photocurrent density of the Fe_2_O_3_ nanowire arrays fabricated at different experimental conditions. Under each temperature, the polished samples showed a higher photocurrent density than that of the unpolished samples, which could be explained by the higher density of the Fe_2_O_3_ nanowire arrays in the polished sample providing more photogenerated carriers, which consequently enhanced the photocurrent density. The samples heated at 600°C with the surface polishing treatment exhibited the highest photocurrent density of 0.9 mA cm^−2^ at 1.23 V versus RHE, which was thrice that of the unpolished sample fabricated at the same temperature. This value was higher than that reported in other literatures [19–21].

**Figure 8.**
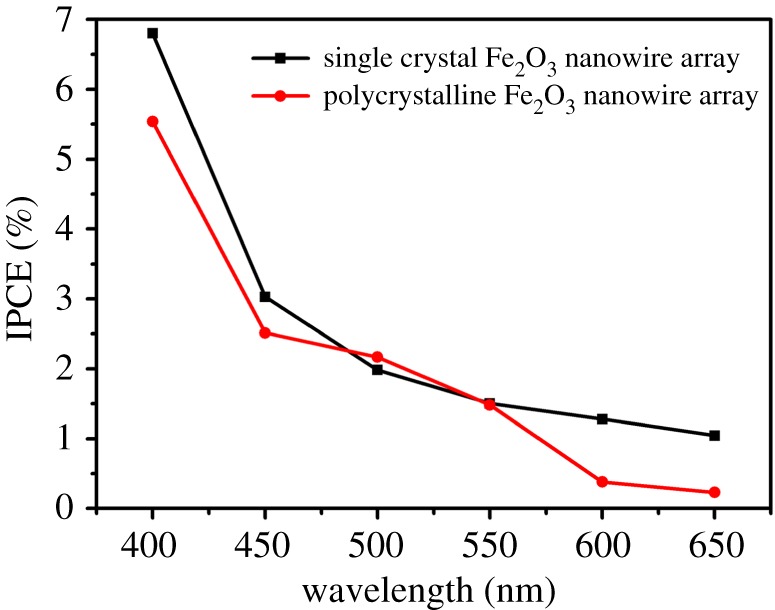
IPCE of the Fe_2_O_3_ nanowire array photoanode at 0.234 V versus Ag/AgCl (1.23 V versus RHE).

Figure 8 presents the IPCE of the nanowire array fabricated at 600°C from the polished sample, which was 6.8% at 400 nm at 1.23 V versus RHE. This value was higher than that of the polycrystalline Fe_2_O_3_ nanowire array that we previously reported (i.e. IPCE of 5.54% at 400 nm) [23]. Moreover, the IPCE value reported herein was relatively high compared with the other undoped Fe_2_O_3_ photoanodes fabricated with metallic substrates, such as undoped Fe_2_O_3_ nanoflakes on an Fe foil with an IPCE of 3.43% at 400 nm [27], a haematite nanotube array on an Fe foil with an IPCE of 3.2% at 400 nm [28], and an Fe_2_O_3_ nanorod array on a Ti plate with an IPCE of 4.8% at 400 nm [29]. It is considered that the high-density Fe_2_O_3_ nanowire array could provide a higher absorption of the light and the single crystal structure is helpful to reduce the recombination of photogenerated electrons and holes happening in the photoanode. However, a nanostructured Fe_2_O_3_ photoanode fabricated on the fluorine-doped tin oxide (FTO) substrate also showed a higher IPCE value. Liao *et al*. [30] reported an IPCE value of 23% at 400 nm. Normally, an FTO substrate is helpful in increasing the photoelectrochemical performance because it could facilitate the electron transport in the photoanode. Therefore, further increasing the IPCE value of the Fe_2_O_3_ nanowire array photoanode is possible using an FTO substrate and surface function with dopant doping.

**Figure 9.**
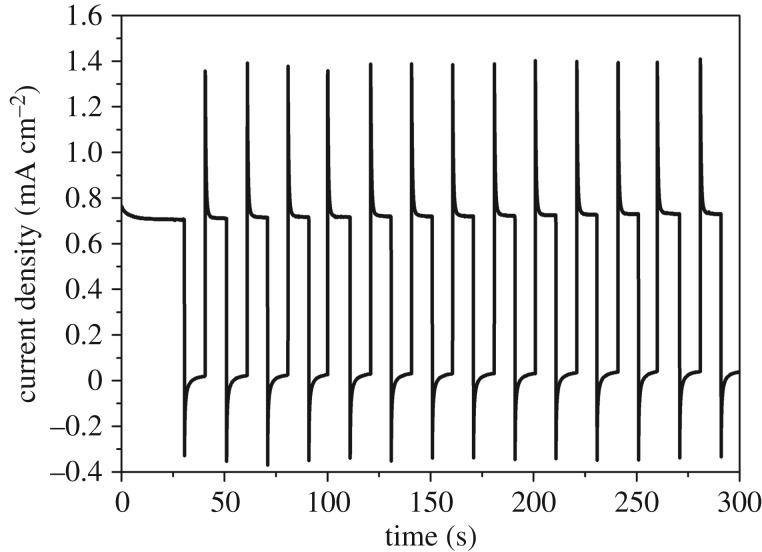
J–t curve of the Fe_2_O_3_ nanowire array photoanode under chopped illumination at a bias of 0.234 V versus Ag/AgCl (1.23 V versus RHE).

Figure 9 illustrates the photocurrent stability. The Fe_2_O_3_ nanowire array photoanode fabricated at 600°C from the polished sample was illuminated with 10 s on/off for 300 s by a chopped light. The result denoted a photocurrent density of 0.75 mA cm^−2^ 1.23 V versus RHE during the illumination. Moreover, the photocurrent of the prepared sample quickly changed with the light switched off, thereby showing good photoresponse properties.

## 4. Mechanism

The fabrication mechanism of the metal oxide nanowire arrays with similar methods was also reported [31,32]. In our previous study, we studied the nanowire growth mechanism based on oxidation-assisted stress-induced atomic diffusion. Tensile stress was generated at the Fe plate surface because of the different molar volumes of Fe_2_O_3_ and Fe. Thus, a stress gradient was generated from the centre of the Fe plate to the Fe_2_O_3_–Fe interface as the driving force for the nanowire growth [23].

**Figure 10.**
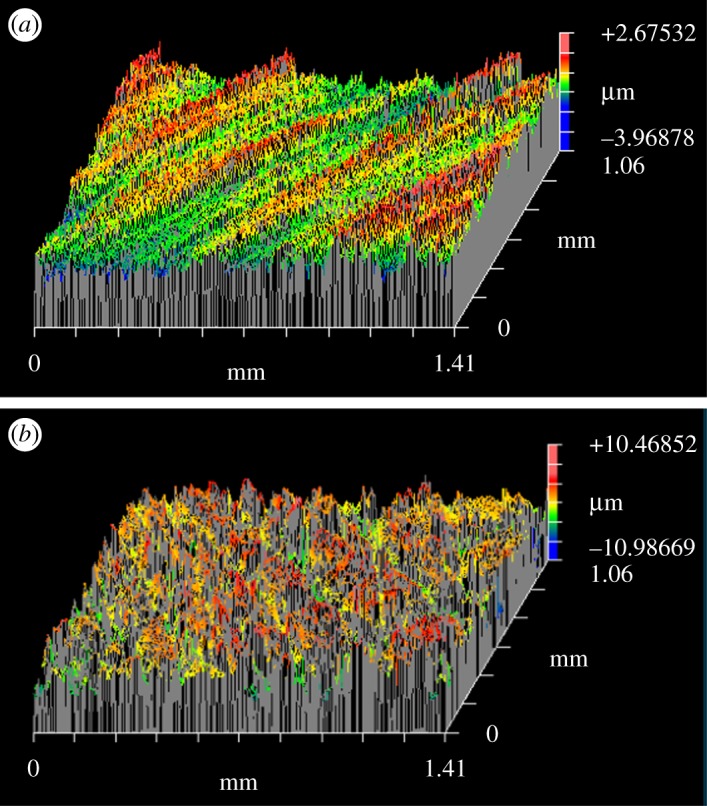
Surface roughness results: (*a*) unpolished sample and (*b*) polished sample.

**Figure 11.**
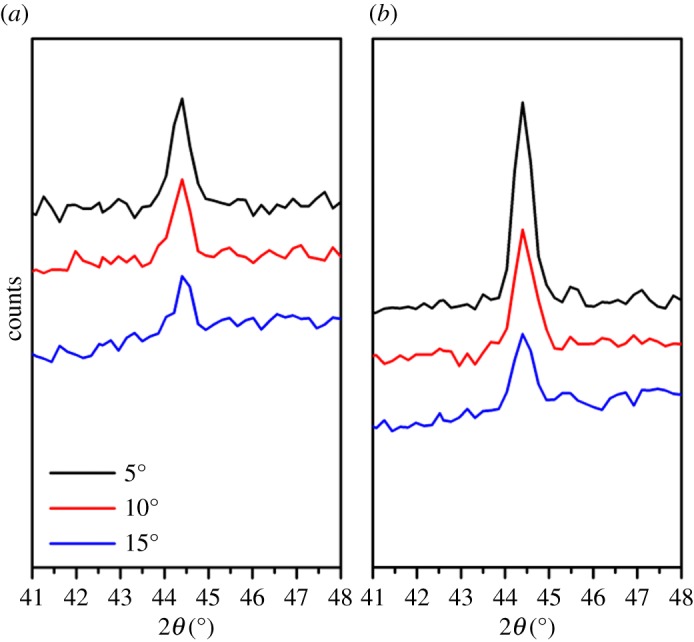
XRD results used for the calculation of residual stress: (*a*) unpolished sample and (*b*) polished sample.

**Figure 12.**
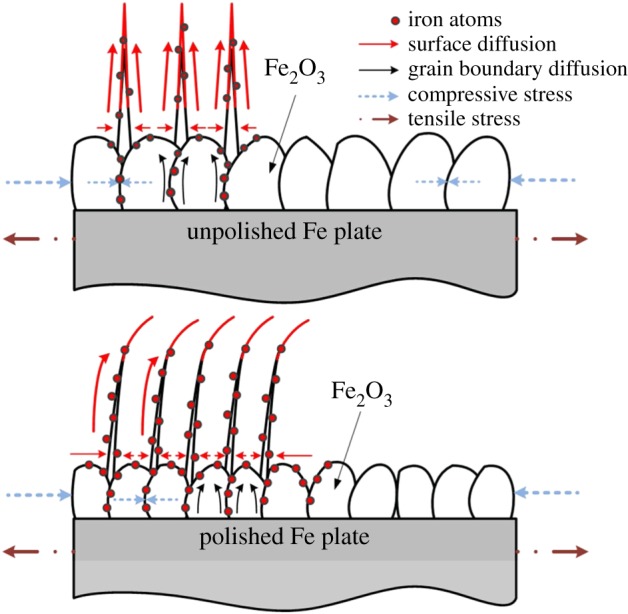
Schematic of the nanowire growth mechanism.

In the present study, the density of the nanowire array fabricated at 600°C on the polished sample was 28.75 wire µm^−2^, which was much higher than that of the nanowire array fabricated from the unpolished sample. Figure 10 shows the measured surface roughness of polished and unpolished samples obtained by a white light interferometer (ZYGO new view 6000). The surface roughness of the polished and unpolished samples is 2.459 µm and 0.452 µm on average, respectively. The surface polishing treatment provided three advantages for the nanowire growth. First, the polishing treatment induced an initial compressive residual stress on the Fe plate surface, which obstructed the volume expansion of the oxide layer formed on the sample surface during the heating process. Therefore, a higher effective vertical stress gradient would occur for the same oxide volume expansion, which induces more and faster diffusion of Fe atoms to the sample surface, thereby increasing the nanowire array density. The residual stress has been measured by XRD and calculated by an XRD analyse software (JADE 6.5), as shown in figure 11. The residual stress is −125.3 Mpa in the polished sample and −28.99 Mpa in the unpolished sample, respectively. Second, the polishing treatment will deform the surface layer of the Fe plate, leading to dislocations and intercrystalline failure at the Fe plate surface. Recrystallization will happen if the polished Fe plate is annealed at a high temperature [33]. During the recrystallization process, the grain size of Fe will decrease and the number of grains will increase, which will finally lead to an increase in the oxide volume expansion and the number of weak spots of the Fe_2_O_3_ layer. Third, the surface polishing treatment increases the roughness of the Fe plate surface, which could enlarge the contact area between the sample surface and the air, thereby increasing the volume of the Fe_2_O_3_ layer on the Fe plate. Compared to that of the unpolished samples, the volume expansion of the Fe_2_O_3_ layer for the polished sample was larger during the heating process, which enhanced the tensile stress at the Fe plate surface and increased the driving force for the Fe atomic diffusion (figure 12). In summary, the surface polishing treatment could provide a larger driving force and increase the number of weak spots, which could eventually increase the nanowire array density.

## 5. Conclusion

In this study, high-density single-crystal Fe_2_O_3_ nanowire arrays were fabricated based on the oxidation-assisted stress-induced atomic diffusion method from the substrate with a surface polishing treatment. The surface polishing treatment could provide a larger driving force and increase the number of weak spots on the sample surface, leading to an increase in the nanowire density. The nanowire array with the highest density of 28.75 wire µm^−2^ was obtained from the polished sample heated at 600°C for 90 min. A photocurrent density of 0.9 mA cm^−2^ at 1.23 V versus RHE and an IPCE of 6.8% at 400 nm were also obtained. The stability test results showed good photocurrent response and stability of the single-crystal Fe_2_O_3_ nanowire photoanode, which indicated a good potential for the solar water splitting application.

## Supplementary Material

Supplementary information figure data

## Data accessibility

The datasets supporting this article have been uploaded as part of the electronic supplementary material.
